# 902 Factors Associated with Burn Center Care for Pediatric Burn Injuries: A Population-based Study

**DOI:** 10.1093/jbcr/iraf019.433

**Published:** 2025-04-01

**Authors:** Eduardo Gus, Teresa To, Joel Fish, Christina Diong, Natasha Saunders

**Affiliations:** The Hospital for Sick Children; The Hospital for Sick Children; The Hospital for Sick Children; Institute for Clinical Evaluative Sciences; The Hospital for Sick Children

## Abstract

**Introduction:**

Burns meeting specific clinical criteria should be managed at specialized burn centres. In our province, from 2003 to 2022, six burn centres provided specialized care for pediatric burns. This study aims to identify factors associated with pediatric burn care at these centres and to assess how well the burn centre referral criteria are adhered to.

**Methods:**

This was a population-based cohort study using linked health administrative datasets. Using hospital discharge records, we identified children (0 to 17 years) with a hospital visit for burn injury between 2003 and 2022. The main exposure was the presence of one or more measurable burn centre referral criteria: 1) burns > 10% TBSA, 2) full thickness burns, 3) burns to special anatomic areas, 4) inhalation, 5) chemical and 6) electrical injuries. We also measured demographic and social vulnerability factors of burned children. The outcome was receipt of treatment at a burn centre. We used standardized differences (SD) to measure differences in management setting by sociodemographic factors and modified Poisson regression to test the association between presence of burn center referral criteria and treatment at a burn centre.

**Results:**

Of 79,782 children and adolescents with burn injuries, 13,531 (17%) were treated at burn centres. Compared to non-burn centres, burn centres treated a higher proportion of children 0–1-year-old (15% vs. 9%, SD 0.19), 1-4 years old (49% vs. 40%, SD 0.20), and urban residents (94% vs. 77%, SD 0.51). No meaningful differences in treatment setting were observed by sex or by other measures of social vulnerability. Children with at least one referral criterion, compared to those with none, had an increased risk of treatment at a burn centre (1+ referral criterion 7,753/34,812 [22%] burns; no referral criteria 5,998/44,970 [13%] burns; adjusted relative risk [aRR] 1.50, 95% CI 1.46 – 1.54). With increasing numbers of burn centre referral criteria, the risk of treatment at a burn centre increased (referent: no burn centre referral criterion; single criterion 5,398/29,768, aRR 1.27, 95% CI 1.23-1.30; two criteria 1,748/4,459, aRR 2.63, 95% CI 2.51-2.75; three or more criteria 387/585, aRR 4.71, 95% CI 4.32-5.15).

**Conclusions:**

Compared to non-burn centres, burn centres treat a higher proportion of young individuals, and urban residents. Treatment at a burn centre was low regardless of the presence of one or more burn centre referral criteria and 1.5 times higher if a burn centre referral criterion was present.

**Applicability of Research to Practice:**

Understanding health system, hospital and clinician barriers and facilitators to aligning burn care delivery with patient needs is important to ensure system efficiency and optimal patient outcomes.

**Funding for the Study:**

This study was funded by a Studentship Award.

